# Effects of Active Upper Limb Orthoses Using Brain–Machine Interfaces for Rehabilitation of Patients With Neurological Disorders: Protocol for a Systematic Review and Meta-Analysis

**DOI:** 10.3389/fnins.2021.661494

**Published:** 2021-06-24

**Authors:** Emília M. G. S. Silva, Ledycnarf J. Holanda, Gustavo K. B. Coutinho, Fernanda S. Andrade, Gabriel I. S. Nascimento, Danilo A. P. Nagem, Ricardo A. de M. Valentim, Ana Raquel Lindquist

**Affiliations:** ^1^Laboratory of Intervention and Analysis of Movement, Department of Physical Therapy, Federal University of Rio Grande do Norte, Natal, Brazil; ^2^Laboratory of Technological Innovation in Health, Federal University of Rio Grande do Norte, Natal, Brazil; ^3^Department of Biomedical Engineering, Federal University of Rio Grande do Norte, Natal, Brazil

**Keywords:** brain-machine interface, upper limb orthosis, upper limb rehabilitation, neurological diseases, independence

## Abstract

**Introduction:** The field of brain–machine interfaces (BMI) for upper limb (UL) orthoses is growing exponentially due to improvements in motor performance, quality of life, and functionality of people with neurological diseases. Considering this, we planned a systematic review to investigate the effects of BMI-controlled UL orthoses for rehabilitation of patients with neurological disorders.

**Methods:** This systematic review and meta-analysis protocol was elaborated according to the Preferred Reporting Items for Systematic Reviews and Meta-Analyses Protocols (PRISMA-P 2015) and Cochrane Handbook for Systematic Reviews of Interventions. A search will be conducted on Pubmed, IEEE Xplore Digital Library, Medline, and Web of Science databases without language and year restrictions, and Patents Scope, Patentlens, and Google Patents websites in English, Spanish, French, German, and Portuguese between 2011 and 2021. Two independent reviewers will include randomized controlled trials and quasi-experimental studies using BMI-controlled active UL orthoses to improve human movement. Studies must contain participants aged >18 years, diagnosed with neurological disorders, and with impaired UL movement. Three independent reviewers will conduct the same procedure for patents. Evidence quality and risk of bias will be evaluated following the Cochrane collaboration by two review authors. Meta-analysis will be conducted in case of homogeneity between groups. Otherwise, a narrative synthesis will be performed. Data will be inserted into a table containing physical description, UL orthoses control system, and effect of BMI-controlled orthoses.

**Discussion:** BMI-controlled orthoses can assist individuals in several routine activities and provide functional independence and sense of overcoming limitations imposed by the underlying disease. These benefits will also be associated with orthoses descriptions, safety, portability, adverse events, and tools used to assess UL motor performance in patients with neurological disorders.

**PROSPERO Registration Number:** CRD42020182195.

## 1. Introduction

Brain–machine interfaces (BMI) are innovative control strategies that use brain activity to control virtual environments with different immersion degrees and external devices, such as orthoses, wheelchairs, exoskeletons, and communication tools (McConnell et al., 2017; Guy et al., 2018). BMI-controlled orthoses are flexible systems composed of mechanical and computational resources that generate complex functional movements (Tanaka et al., 2014; McConnell et al., 2017) using brain activity to assist people with different neurological conditions (Lee et al., 2019).

Several age-related molecular and physiological changes or abnormal neuroplasticity may be associated with neurological disorders (Mohammadi, 2016; Carroll, 2019). Therefore, previous researchers developed technologies to stimulate and favor patient's rehabilitation (i.e., improve active participation and involvement in the rehabilitation process) using control commands generated by brain signals (Picha and Howell, 2018).

Consequently, these orthoses can be implemented as new therapeutic strategies based on specific tasks, enhancing rehabilitation results (Picha and Howell, 2018; Baniqued et al., 2021) and favoring functionality during daily tasks (Carvalho et al., 2019; Yurkewich et al., 2020; Baniqued et al., 2021). Although several resources (e.g., passive orthoses, exoskeletons, and gloves) may lead to motor and functional gains in patients with neurological disorders, BMI-controlled orthoses can be integrated into sensory stimulus to improve motor performance (McConnell et al., 2017), motor function, and cortical excitability (Hortal et al., 2015; Bockbrader, 2019; Kapsalyamov et al., 2019). Unlike several resources used to achieve motor and functional gains in patients with neurological disorders (e.g., passive orthoses, exoskeletons, and gloves), BMI-controlled orthoses can be integrated into sensory stimulus to improve motor performance (McConnell et al., 2017). Development and application of BMI-activated systems have been growing in recent years, mainly due to rehabilitation benefits. Clinical and neurophysiological changes were observed in the upper limb (UL) of post-stroke survivors after neurofeedback training with BMI (Carvalho et al., 2019). Also, systematic reviews evaluated the impact of robotic hand systems controlled by BMI on fine motor skills associated with technical specifications of devices (Baniqued et al., 2021).

Previous research observed a trend in assistive home-based technologies for rehabilitation, highlighting their portability potential (Chu and Patterson, 2018; Ramos-Murguialday et al., 2019). Thus, it is necessary to insert safety mechanisms into portable and easy-to-handle orthoses to favor residential environments. Chu and Patterson (2018) emphasized that <50% of studies on the topic addressed portability, while only 27% addressed malleable hand orthoses safety to improve movements in patients with movement disorders.

Here, we describe the protocol for the first systematic review and meta-analysis on BMI-based UL orthosis for patients with neurological conditions, considering patents published in the last 10 years and all articles published to date. Thus, this review will explore research questions about control systems, physical orthoses description, BMI types, and their effects in patients with neurological diseases to achieve the sub-aims of this study (Table 1). The sub-aims will be as follows: (1) describe the control systems and physical descriptions of BMI-based orthoses, as well as their ability to be used in home-based rehabilitation; (2) describe the BMI types that are used on BMI-based orthoses; (3) determine whether specific BMI types favor better motor function performance; (4) describe the tools used to evaluate the effectiveness of BMI-based orthoses and the context of the assessment (task description, duration, frequency, and number of repetitions); (5) determine the effectiveness of BMI-based orthoses; and (6) determine whether patented BMI-based orthoses had their reported results in scientific articles.

**Table 1:** Research questions.

**Sub-aims**	**List of questions**
Describe the control systems and physical descriptions of BMI-based orthoses, as well as their ability to be used in home-based rehabilitation	• What are the control systems of BMI-based orthoses? • What are the physical descriptions of BMI-controlled orthoses? • Have been these orthoses tested during home-based rehabilitation? • Do BMI-based orthoses have secure and portable systems to ensure their use in home-based rehabilitation strategies for people with neurological disorders?
Describe the BMI types that are used on BMI-based orthoses	• What BMI types are used on BMI-based orthoses?
Determine whether specific BMI types favor better motor function performance	• What BMI types can favor better motor function performance?
Describe the tools used to evaluate the effectiveness of BMI-based orthoses and the context of the assessment (task description, duration, frequency, and number of repetitions)	• What were the tools used to evaluate the effectiveness of BMI-based orthoses? • How was the assessment done, considering the description of the task, duration, frequency, and number of repetitions?
Determine the effectiveness of BMI-based orthoses	• What were the benefits found in individuals who have used BMI-based orthoses?
Determine whether patented BMI-based orthoses had their reported results in scientific articles	• How many registered patents had their results about control systems and physical descriptions reported in articles? • What are the control systems and physical descriptions of BMI-based orthoses registered in patents?

Thus, this review aimed to analyze the effects of active UL orthoses using BMI for rehabilitation of patients with neurological diseases. Information collected will guide future research related to new orthoses development, while information on physical description and BMI types will help professionals during the rehabilitation of patients with neurological diseases.

## 2. Materials and Methods

This systematic review protocol was registered on PROSPERO (registration no. CRD42020182195). Preferred Reporting Items for Systematic Reviews and Meta-Analyses for Protocols 2015 (PRISMA-P 2015) will be used to provide comprehensive guidance to prepare and report information. Search procedures were ultimately reported and reproducible (Moher et al., 2010; Rethlefsen et al., 2021). In this perspective, PRISMA-P 2015 (Moher et al., 2010) (see Supplementary File 1) and its extension (Rethlefsen et al., 2021) (see Supplementary File 2) supplies standard guidelines to elaborate the systematic review. The review will also be performed following the Cochrane Handbook for Systematic Reviews of Interventions (Higgins et al., 2019).

### 2.1. Eligibility Criteria

We established eligibility criteria according to PICOS strategy (Population, Intervention, Comparison, Outcomes, and Study). Patents for BMI-controlled orthoses will be selected according to publication year (registered in the last 10 years) and language (Portuguese, English, Spanish, French, and German).

#### 2.1.1. Types of Participants

We will select studies in which orthosis effectiveness was evaluated in humans with acute or chronic diagnosis of any neurological disease, regardless of severity and impaired UL movement. Participants must be over 18 years of age due to differences in neuroplasticity mechanisms (Mohammadi, 2016).

#### 2.1.2. Types of Interventions

We will select studies that used BMI-based UL orthosis. Besides, studies involving only games will be excluded.

#### 2.1.3. Types of Comparisons

We will select studies comparing:

- BMI-based active orthosis vs. passive orthosis;
- BMI-based active orthosis alone or associated with other therapy for UL motor function rehabilitation, such as physical therapy or occupational therapy;
- BMI-based active orthosis vs. no therapy.

#### 2.1.4. Types of Outcomes

##### 2.1.4.1. Primary Outcomes

Primary outcomes will be clinical effectiveness of BMI-controlled UL orthoses on motor function of people with neurological disorders using biomechanical assessment tools or qualitative or quantitative validated functional scales for UL motion (COSMIN) (Mokkink et al., 2012).

##### 2.1.4.2. Secondary Outcomes

Secondary outcomes will include physical description and control system features of orthoses, safety, portability, dropouts, adherence, and adverse events, such as pain and skin irritation.

#### 2.1.5. Types of Studies

We will include randomized controlled trials (RCTs) and quasi-experimental studies because the primary aim of this review. Furthermore, non-RCTs will be inserted, including observational studies to describe control system features and physical descriptions of orthoses.

We will also include patents that registered BMI-controlled UL orthoses to investigate the physical description of those that have already been developed and registered.

### 2.2. Search Methods for Study Selection

#### 2.2.1. Electronic Searches

A search strategy will be conducted in the following electronic databases to select potential articles: Pubmed, IEEE Xplore Digital Library, Medline, and Web of Science. We will also conduct a similar search on Patents Scope, Patentlens, and Google Patents websites.

#### 2.2.2. Searching Other Resources

We will verify the gray literature (i.e., theses, dissertations, conference papers, and reference articles) and search the following clinical trials websites:

- USA National Institutes of Health Ongoing Trials Register ClinicalTrials.gov (www.clinicaltrials.gov/);
- Brazilian Clinical Trials Registry/Registro Brasileiro de Ensaios Clínicos (ReBEC) (https://ensaiosclinicos.gov.br/).

##### 2.2.2.1. Search Strings

Searches will be refined using strings related to body segment, physical rehabilitation, orthosis, and BMI (Table 2). All search strategies will be documented (see Supplementary File 3).

**Table 2:** Keywords that will be used in search strategies.

**Aspects**	**Matching keywords**
Body segment	• Hand, Elbow, Wrist, Shoulder, Forearm, Arm, Fingers, Upper Extremity, Upper Limb.
Orthosis	• Orthosis, Orthoses, Active Orthosis, Active Orthoses, Exoskeleton, Orthotic Device, Orthosis Device, Orthoses Device, Robotic Device, Robotic, Wearable Robot, Exosuit, Wearable Orthoses, Wearable Orthosis, Wearable Assistive Robots, Wearable Exosuit.
Physical rehabilitation	• Physical Rehabilitation, Motor Rehabilitation, Physical Medicine, Telerehabilitation.
Brain–machine interface	• Brain–Machine Interface, Brain–Computer Interface, User–Computer Interface, Virtual Systems, Human–Machine Interface, Man–Machine Interface.

#### 2.2.3. Data Management

Two independent reviewers (ES and LH) will select articles, and the other three (GC, FA, and GN) will select patents. Study selection process will be summarized in the PRISMA flowchart (see Supplementary File 4) (Moher et al., 2010). In addition, review articles will be excluded. Thus, we will extract information of the included articles and perform the meta-analysis, if possible. All discrepancies will be resolved by consensus in team sessions.

#### 2.2.4. Data Collection Process

A data extraction table will be created, and articles and patents will be independently assessed and extracted by two teams.

#### 2.2.5. Data Items

The following data will be extracted from each article and patent: physical description of UL orthoses (assisted motion, assisted body segment, portability, safety, sensor, total degree of freedom, and actuators), device operation, control system characteristics (BMI type, electrodes number, control strategy, and supporting feedback), participant characteristics (sample size, clinical diagnosis, time of diagnosis, age, and sex), and orthosis evaluation (assessment, frequency, duration, and task description).

### 2.3. Dealing With Missing Data

We will contact researchers to verify key study characteristics and obtain missing numerical data when possible (e.g., when a study is available as abstract only). In case of no response and considering that missing data will enhance bias, we will conduct a sensitivity analysis to determine the impact of including these studies. We plan to perform the following sensitivity analyses to ensure results are robust and meaningful. We will repeat the analysis, excluding studies with high risk of bias (non-blinded trials, questionable randomization methods, bias related to control group management).

### 2.4. Risk of Bias in Individual Studies

Two review authors (ES and LH) will use the Cochrane Risk of bias tool (Higgins et al., 2003), considering the included study types. Any disagreements will be solved by discussing with another review author (AL). GRADE system will evaluate the following intervention bias in RCTs studies: selection bias, performance bias, detection bias, attrition bias, reporting bias, and other biases (Higgins et al., 2003). However, risk of bias in non-randomized studies will be evaluated using Risk of Bias in Non-randomized Studies of Interventions (ROBINS-I), which divides bias into seven domains: confounding, participant selection, intervention classification, deviations from intended interventions, missing data, outcome measurement, and selection of reported result (Higgins et al., 2003).

Risk of bias assessment will also consider the influence of unblinding. Thus, high risk of bias will be assigned if participants and personnel are not blinded and we judge results could be influenced by participants and personnel knowledge regarding the treatment provided (Higgins et al., 2003).

We will grade trials as low, high, or unclear risk of bias (“unclear” will indicate lack of information to judge the presence of bias or uncertain risk of bias). If risk of bias is unclear due to insufficient information, we will attempt to contact authors to obtain further information and categorize risk of bias.

### 2.5. Subgroup Analysis and Investigation of Heterogeneity

We will perform subgroup analyses to determine the clinical effectiveness of BMI-controlled orthosis as rehabilitation tool for people with neurological disorders. We will conduct the following subgroup analyses: study designs (randomized vs. non-randomized), participant characteristics (types of clinical diagnosis), and BMI types.

Heterogeneity across studies will be evaluated using I^2^ (Higgins and Altman, 2011) and interpreted as (Deeks et al., 2019): 0 and 40%, might not be important; 30–60%, may represent moderate heterogeneity; 50–90% may represent substantial heterogeneity; 75–100%, considerable heterogeneity. Statistically significance will be considered if I^2^ statistic is greater than 50% or chi-squared *P*-value is < 0.10 (RevMan, 2014). Sources of heterogeneity will be reported, and possible causes will be investigated using subgroup analysis. We will evaluate heterogeneity between treatment tools before performing meta-analysis, focusing on the distribution of individuals in subgroup analysis.

### 2.6. Assessment of Methodological Quality

We will elaborate a “Summary of findings” table, divided into orthoses physical description and control system, and outcomes (motor function, safety, portability, and adverse events). Evidence quality will be assessed following the GRADE system (Guyatt et al., 2011), which grades evidence in four levels: high, moderate, low, and very low. For high-quality evidence, randomized, double-blinded studies with no selection bias will be considered, while observational studies with methodological limitations will be considered impartial.

### 2.7. Statistical Analysis and Data Synthesis

In case of homogeneity among studies, a meta-analysis will be conducted using Review Manager 5 (Collaboration, 2014) or an updated version, if available. We will consider similar outcome measures between studies, clinical characteristics regarding type and disease severity, BMI type, methods (i.e., treatment type and length), and study design.

In cases of methodological heterogeneity and different outcome measures, a narrative synthesis will be conducted. We will determine the number and percentage of articles and patents included in each cluster, such as BMI type, participant characteristics, and country of origin of first authors. Depending on the number of articles and patents within each cluster, clustering may be performed for more levels or more variables or both, leading to a set of nested clusters. This result will be presented in a bubble plot, graph, or table. Trend analysis will be used to identify associations and present the research progress based on several variables.

## 3. Discussion

BMI-controlled orthoses can assist in routine activities, provide functional independence, and overcome limitations imposed by the disease (Picha and Howell, 2018). This review will provide a literature overview on BMI as UL orthosis control strategy for motor rehabilitation of patients with neurological disorders, considering their impact on functionality and motor function, and associating with adverse events, safety, portability, physical descriptions, and control system features.

To our knowledge, this will also be the first review to extract data from orthoses in the patent process and articles, offering guidance on existing designs in this area. Moreover, this knowledge will guide professionals regarding improvements in this type of technology to motivate rehabilitation based on patient needs.

Although we do not yet know the potential benefits or preferences, our findings will provide guidance on clinical and long-term benefits to clients after neurological injury and impairment. In addition, devices should be hybrid, consider functional disability of each neurological disorder, and include feedback, safety, and portable mechanisms to improve user benefits and provide a user-centered therapy.

## Data Availability Statement

The original contributions presented in the study are included in the article/Supplementary Material, further inquiries can be directed to the corresponding author/s.

## Author Contributions

ES and LH conceptualized the protocol and established eligibility criteria, search strategy, and data extraction framework, which was then further developed with the input of team members. ES, LH, GC, FA, and GN wrote the protocol. AL, DN, and RV provided expert advice on this review subject. All authors made substantial contributions to this protocol, read, and approved the final version.

## Conflict of Interest

The authors declare that the research was conducted in the absence of any commercial or financial relationships that could be construed as a potential conflict of interest.

## Supplementary Material

Supplementary File 1 PRISMA-P 2015 checklist.

Supplementary File 2 Updated of PRISMA-P checklist.

Supplementary File 3 Search strategies.

Supplementary File 4 PRISMA flowchart.
