# Supplementary material for: Effects of Active Upper Limb Orthoses Using Brain–Machine Interfaces for Rehabilitation of Patients With Neurological Disorders: Protocol for a Systematic Review and Meta-Analysis
Source: Front Neurosci. 2021 Jun 24;15:661494. doi: 10.3389/fnins.2021.661494 (PMC8264786; doi:10.3389/fnins.2021.661494)
Supplement: Supplementary File 3 — Search strategies. [file Table_3.doc]

**ADDITIONAL FILE 3 SEARCH STRATEGIES**

**Pubmed**

((((((((((((((((((((((((((((((hand) OR elbow) OR wrist) OR shoulder) OR forearm) OR arm) OR fingers) OR upper extremity) OR upper limb) AND physical Rehabilitation OR motor rehabilitation) OR physical medicine) OR telerehabilitation) AND orthoses) OR orthosis) OR active orthoses) OR active orthosis) OR exoskeleton) OR orthotic device) OR orthosis device) OR orthoses device) OR robotic device) OR robotic) OR wearable robot) OR exosuit) OR wearable orthoses) OR wearable orthosis) OR wearable assistive robots) OR wearable exosuit) AND brain machine interface) OR user-computer interface) OR virtual systems) OR human machine interface) OR man machine interface)))))))))))))))))))))))))))))))

**IEEE**

(hand OR elbow OR wrist OR shoulder OR forearm OR arm OR fingers OR upper extremity OR upper limb) AND (physical rehabilitation OR motor rehabilitation OR physical medicine OR telerehabilitation) AND (orthoses OR orthosis OR active orthosis OR active orthoses OR exoskeleton OR orthotic device OR orthosis device OR orthoses device OR robotic device OR robotic OR wearable robot OR exosuit OR wearable orthoses OR wearable orthosis OR wearable assistive robots OR wearable exosuit) AND (brain machine interface OR user-computer interface OR virtual systems OR human machine interface OR man machine interface)

**Medline**

1. hand/ or elbow/ or wrist/ or shoulder/ or forearm/ or arm/ or fingers/ or upper extremity/ or upper limb/

2. physical rehabilitation/ or motor rehabilitation/ or physical medicine/ or telerehabilitation/

3. orthosis/ or orthoses/ active orthosis/ or active orthoses/ or exoskeleton/ or orthotic device/ or orthosis device/ or orthoses device/ or robotic device/ or robotic/ or wearable robot/ or exosuit/ or wearable orthoses/ or wearable orthosis/ or wearable assistive robots/ or wearable exosuit/

4. brain machine interface/ or brain computer interface/ or user-computer interface/ or virtual systems/ or human machine interface/ or man machine interface/

5. and/1-4

**Web of Science**

#1 TI =(hand OR elbow OR wrist OR shoulder OR forearm OR arm OR fingers OR upper extremity OR upper limb)

#2 TI = (physical rehabilitation OR motor rehabilitation OR physical medicine OR telerehabilitation)

#3 TI = (orthoses OR orthosis OR active orthoses OR active orthosis OR exoskeleton OR orthotic device OR orthosis device OR orthoses device OR robotic device OR robotic OR wearable robot OR exosuit OR wearable orthoses OR wearable orthosis OR wearable assistive robots OR wearable exosuit)

#4 TI = (brain machine interface OR user-computer interface OR virtual systems OR human machine interface OR man machine interface))

# 5 = #1 AND #2 AND #3 AND #4 AND

#6 TS =(hand OR elbow OR wrist OR shoulder OR forearm OR arm OR fingers OR upper extremity OR upper limb)

#7 TS = (physical rehabilitation OR motor rehabilitation OR physical medicine OR telerehabilitation)

#8 TS = (orthoses OR orthosis OR active orthoses OR active orthosis OR exoskeleton OR orthotic device OR orthosis device OR orthoses device OR robotic device OR robotic OR wearable robot OR exosuit OR wearable orthoses OR wearable orthosis OR wearable assistive robots OR wearable exosuit)

#9 TS = (brain machine interface OR user-computer interface OR virtual systems OR human machine interface OR man machine interface))

#10 = #6 AND #7 AND #8 AND #9 AND

#11= #5 AND #11

**Patents Scope**

((hand OR elbow OR wrist OR shoulder OR forearm OR arm OR fingers OR upper extremity OR upper limb)) AND ((physical rehabilitation OR motor rehabilitation OR physical medicine OR telerehabilitation)) AND ((orthoses OR orthosis OR active orthoses OR active orthosis OR exoskeleton OR orthotic device OR orthosis device OR orthoses device OR robotic device OR robotic OR wearable robot OR exosuit OR wearable orthoses OR wearable orthosis OR wearable assistive robots OR wearable exosuit)) AND ((brain machine interface OR user-computer interface OR virtual systems OR human machine interface OR man machine interface))

**Patentlens**

((hand OR elbow OR wrist OR shoulder OR forearm OR arm OR fingers OR upper extremity OR upper limb)) AND ((physical rehabilitation OR motor rehabilitation OR physical medicine OR telerehabilitation)) AND ((orthoses OR orthosis OR active orthoses OR active orthosis OR exoskeleton OR orthotic device OR orthosis device OR orthoses device OR robotic device OR robotic OR wearable robot OR exosuit OR wearable orthoses OR wearable orthosis OR wearable assistive robots OR wearable exosuit)) AND ((brain machine interface OR user-computer interface OR virtual systems OR human machine interface OR man machine interface))

**Google Patents**

((hand OR elbow OR wrist OR shoulder OR forearm OR arm OR fingers OR upper extremity OR upper limb)) AND ((physical rehabilitation OR motor rehabilitation OR physical medicine OR telerehabilitation)) AND ((orthoses OR orthosis OR active orthoses OR active orthosis OR OR exoskeleton OR orthotic device OR orthosis device OR orthoses device OR robotic device OR robotic OR wearable robot OR exosuit OR wearable orthoses OR wearable orthosis OR wearable assistive robots OR wearable exosuit)) AND ((brain machine interface OR user-computer interface OR virtual systems OR human machine interface OR man machine interface))
